# THE EFFECTIVENESS OF A CASE MANAGEMENT PROGRAM FOR FAMILY CAREGIVERS OF PERSONS WITH MCI OR EARLY STAGE DEMENTIA

**DOI:** 10.1093/geroni/igad104.1518

**Published:** 2023-12-21

## Abstract

This study examined the effectiveness of a case management program for family caregivers of persons with mild cognitive impairment, early to mild dementia. Using a quasi-experimental study design, we recruited family caregiver and care recipient dyads from memory clinics in Taiwan. We randomly assigned the dyads into experimental groups (n= 38 dyads) or control groups (n= 33 dyads). The experimental group participants received case manager services over a four-month period, while those in the control group received general health education. Data were collected at three time points: baseline, the 4th and 6th months for both groups. The results of the generalized estimating equation revealed that in the fourth month, the experimental group exhibited a declined depression (β = −3.74, p < .001) and increased use of social resources (β = 1.014, p = .045). In the sixth month, the depression of caregivers declined further (β = −3.186, p = .002), whereas their use of social resources showed no significant difference. This study illuminates the feasibility and efficacy of using a case management model to support caregivers encountered in memory clinics. Findings are promising to suggest the need to call for more education and supports available at clinics to prepare and assist caregivers who have a relative of at the early stage of cognitive impairment or dementia.

